# Genome profiling of chronic myelomonocytic leukemia: frequent alterations of *RAS *and *RUNX1 *genes

**DOI:** 10.1186/1471-2407-8-299

**Published:** 2008-10-16

**Authors:** Véronique Gelsi-Boyer, Virginie Trouplin, José Adélaïde, Nicola Aceto, Virginie Remy, Stephane Pinson, Claude Houdayer, Christine Arnoulet, Danielle Sainty, Mohamed Bentires-Alj, Sylviane Olschwang, Norbert Vey, Marie-Joëlle Mozziconacci, Daniel Birnbaum, Max Chaffanet

**Affiliations:** 1 Centre de Recherche en Cancérologie de Marseille, Laboratoire d'Oncologie Moléculaire, UMR891 Inserm, Institut Paoli-Calmettes, Marseille, France; 2 Département de BioPathologie, Institut Paoli-Calmettes, Marseille, France; 3 Université de la Méditerranée, Marseille, France; 4 Friedrich Miescher Institute for Biomedical Research, Basel, Switzerland; 5 Service de génétique clinique et moléculaire, Hôpital Edouard Herriot, Lyon, France; 6 Laboratoire de génétique constitutionnelle, Institut Curie, Paris, France; 7 Département d'Hématologie, Institut Paoli-Calmettes, Marseille, France

## Abstract

**Background:**

Chronic myelomonocytic leukemia (CMML) is a hematological disease close to, but separate from both myeloproliferative disorders (MPD) and myelodysplastic syndromes and may show either myeloproliferative (MP-CMML) or myelodysplastic (MD-CMML) features. Not much is known about the molecular biology of this disease.

**Methods:**

We studied a series of 30 CMML samples (13 MP- and 11 MD-CMMLs, and 6 acutely transformed cases) from 29 patients by using Agilent high density array-comparative genomic hybridization (aCGH) and sequencing of 12 candidate genes.

**Results:**

Two-thirds of samples did not show any obvious alteration of aCGH profiles. In one-third we observed chromosome abnormalities (e.g. trisomy 8, del20q) and gain or loss of genes (e.g. *NF1*, *RB1 *and *CDK6*). *RAS *mutations were detected in 4 cases (including an uncommon codon 146 mutation in *KRAS*) and *PTPN11 *mutations in 3 cases. We detected 11 *RUNX1 *alterations (9 mutations and 2 rearrangements). The rearrangements were a new, cryptic inversion of chromosomal region 21q21-22 leading to break and fusion of *RUNX1 *to *USP16*. *RAS *and *RUNX1 *alterations were not mutually exclusive. RAS pathway mutations occurred in MP-CMMLs (~46%) but not in MD-CMMLs. *RUNX1 *alterations (mutations and cryptic rearrangement) occurred in both MP and MD classes (~38%).

**Conclusion:**

We detected RAS pathway mutations and RUNX1 alterations. The latter included a new cryptic *USP16-RUNX1 *fusion. In some samples, two alterations coexisted already at this early chronic stage.

## Background

Chronic myelomonocytic leukemia (CMML) is a heterogeneous hematopoietic disease currently classified by the WHO organization as an entity close to, but separate from both myeloproliferative disorders (MPD) and myelodysplastic syndromes. CMML is included in the category of MPD/MDS diseases and defined by persistent peripheral monocytosis greater than 1 × 10^9^/L, fewer than 20% blasts in the blood or bone marrow (BM), and BM dysplasia in one or more myeloid lineage. Because the blast number is a prognostic factor, CMML is divided in two types: type 1 with fewer than 5% in blood and 10% blasts in BM, and type 2 between 5 and 19% in blood or 10 and 19% in BM [1,2].

The problem of CMML resides in its classification and in the clinical and/or biological relevance of separating the proliferative and dysplastic presentations. The FAB system has recommended a division of CMML in two classes upon leucocyte count: leucocytosis < 13 × 10^9^/L defines CMML as MDS-like (MD-CMML) and leucocytosis > 13 × 10^9^/L as MPD-like (MP-CMML) [2]. The two classes have been variably associated with prognosis and their distinction is a matter of debate [3-7]. This reflects that, except in few imatinib-sensitive cases with PDGFRB alterations, the pathogenesis of CMML is poorly understood. Consequently, the definition and therapy of CMML remain unsatisfactory.

To better understand CMML and improve its classification we have studied the genome of a series of CMML samples by using genome-wide, high-density array-comparative genomic hybridization (aCGH) and DNA sequencing of candidate genes.

## Methods

### Patients and samples

A consecutive series of 30 BM samples were collected from 29 patients including 24 CMMLs and 6 acute transformations of CMML (AT-CMML). Patients were newly diagnosed or were known for hematopoietic disease and the therapeutic impact was evaluated every 3 months. Three patients (3, 52, 90) had received prior chemotherapy for an independent solid tumor. One had an 11q inversion and one had a t(1;3). Clinical and biological data of the 30 samples are presented in Additional file 1. Cytology and phenotype were assessed by three specialists (VGB, CA, DS). Nucleic acids extraction was done as described [8]. Gene expression profiles for the cases with available RNA have been reported [8]. The patients all signed an informed consent. The project and collection of samples were reviewed by the independent scientific review board of the Paoli-Calmettes Institute (COS), in accordance with current regulations and ethical concerns.

### Array comparative genomic hybridization (aCGH)

Genomic imbalances were analyzed by aCGH using 244 K CGH Microarrays (Hu-244A, Agilent Technologies, Massy, France) as previously described [9,10]. The resolution is up to 6 kb. Scanning was done with Agilent Autofocus Dynamic Scanner (G2565BA, Agilent Technologies). Data analysis was made as previously described [10,11] and visualized with CGH Analytics 3.4 software (Agilent Technologies). Extraction data (log_2 _ratio) was done with CGH analytics while normalized and filtered log_2 _ratio were obtained from ≪ Feature extraction ≫ software (Agilent Technologies). Copy number changes were characterized as reported [9,10].

The *RUNX1 *gene map established within Mb scale was extracted from the build 36.1 from NCBI (March 2006 version) while its sequence (Ensembl Transcript ID ENST00000300305) was extracted from Ensembl database , which is based on the Ensembl release 48 – Dec 2007 assembly of the human genome. Genomic profile was established with CGH analytics^® ^software (Agilent Technologies), from centromere to telomere, within the genomic intervals [28.0–30.5 Mb] and [33.8–36.3 Mb] of the short arm of the chromosome 21 (hg17 human genome mapping; build 35 from NCBI, May 2004 version).

### DNA sequencing

Somatic mutations of *BRAF, JAK2, HRAS, KRAS, NRAS, NF1, RAF1, RB1, RUNX1, SOS1, SPRED1 *and *STK11 *genes were searched by sequencing exons and consensus splicing sites after PCR amplification of genomic DNA (Additional file 2). Most PCR amplifications were done in a total volume of 25 μl PCR mix containing at least 10 ng template DNA, Taq buffer, 200 μmol of each deoxynucleotide triphosphate, 20 pmol of each primer and 1 unit of Hot Star Taq (Qiagen). PCR amplification conditions were as follows: 95°C 10 min; 95°C 30 sec, variable temperature 30 sec, 72°C 45 sec for 30 cycles; 72°C 10 min. PCR products were purified using Millipore plate MSNU030.

Two microliters of the purified PCR products were used for sequencing using the Big Dye terminator v1.1 kit (Applied Biosystems). After G50 purification, sequences were loaded on an ABI 3130XL automat (Applied Biosystems). The sequence data files were analyzed using the SeqScape software (Additional file 2) and all mutations were confirmed on an independent PCR product.

### PCR detection of RUNX1 alterations

The *USP16-RUNX1 *gene fusion was detected by using nested PCR amplification of retrotranscribed mRNA (RT-PCR) from BM cells of the patients as previously described [12]. Wild-type and fusion transcripts were amplified using *RUNX1 *and *USP16 *primer sequences (Additional file 3). PCR products were visualized on agarose gel with ethidium bromide, and sequenced.

## Results

### Three types of aCGH profiles in CMML

**Figure 1:**
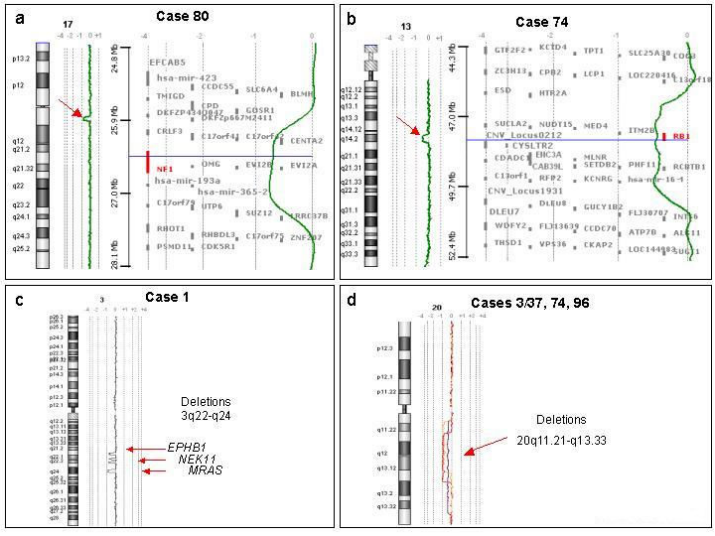
**Examples of aCGH profiles**. A: aCGH profile of chromosome 17 in case 80. Red arrow shows deletion including *NF1*. B: aCGH profile of chromosome 13 in case 74 showing *RB1 *deletion. For a and b, a zoom of the region is shown to the right of the profiles. C: aCGH profile of chromosome 3 in case 1 showing a series of deletions at 3q. D: aCGH profiles of chromosome 20 in cases 3, 37, 74 and 96 (3 and 37 are from the same patient).

Using genome-wide, high-density arrays we established the aCGH profiles of 30 samples from 29 patients, comprising 24 CMMLs and 6 AT-CMMLs. Examples of profiles are shown in Figure 1 and results are summarized in Table 1. Three main types of profiles were observed. Type 1 profiles showed gains or losses visible on the karyotype and affecting large regions of the genome, such as trisomy 8 (10%: cases 5, 12 and 88), deletions of part of the 20q arm (10%: cases 3, 74, and 96), or deletion (case 106) or complex rearrangements of chromosome 7 (case 3). Type 2 profiles showed rare and limited gains or losses that affected few or single genes such as deletions encompassing *NF1 *at 17q11 (case 80), *RB1 *at 13q14 (case 74), *RUNX1 *at 21q21 (case 88), *CALN1 *at 7q11 (case 12), amplification of 7q21 including the *CDK6 *gene (case 3) or a series of short deletions on the 3q arm (case 1). A surprising deletion of the *MYC *locus was observed in case 106. The type 3 profile was said "normal-like" since no obvious alteration was detected. It occurred in two-thirds of the cases.

**Table 1:** Molecular features of the 30 studied CMML

**No**	**Diagnosis**	**Karyotype**	**Array-CGH**	**H, K, NRAS**	**NF1**	**PTPN11 E3, 13**	**RUNX1**	**SOS1 E7-11**	**USP16-RUNX1 rearrangement**
**1**	CMML 2	46, XY [20]	3q22-24 losses (EPHB1, NEK11, MRAS...)	no	nd	**p.Asp61Tyr**	**p.Pro425Leu**	**p.Leu569Val**	nd
**5**	CMML 2	47, XX, +8 [20]	Tri 8	no	nd	**p.Ala72Thr**	**Splicing defect**	no	nd
**6**	CMML 2	46, XY [20]	3p23 loss (GLB1, CRTAP)	no	nd	no	**p.Arg166X**	no	nd
**7**	CMML 1	46, XX [20]	normal-like	no	nd	no	no	no	nd
**12**	CMML 1	47, XX, +8 [20]	Tri 8, 7q11 loss (CALN1)	**N- p.Gly12Asp**	nd	no	**p.Tyr377LeufsX223**	no	nd
13	CMML 1	46, XY [20]	normal-like	no	nd	no	no	no	nd
15	CMML 1	46, XY [20]	normal-like	no	nd	no	**p.Gly50GlnfsX4**	no	nd
**51**	CMML 1	46, XY [20]	normal-like	no	nd	no	no	no	no
**72**	CMML1	46, XY [20]	7p21 gain (AHR)	no	nd	no	no	no	no
**78**	CMML 1	45, X, -X?c [20]	X loss	**N- p.Gly12Asp**	nd	no	no	no	no
**79**	CMML 1	46, XY [20]	normal-like	**K- p.Gly12Ser**	p. =	no	no	no	no

2	CMML 1	46, XY [20]	normal-like	nd	nd	no	no	no	nd
95	CMML 1	46, XX [20]	normal-like	no	nd	no	no	no	no
75	CMML 1	46, XY [20]	normal-like	no	nd	no	no	no	no
**89**	CMML 1	46, XY [20]	normal-like	**K- p.Ala146Val**	nd	no	no	no	no

**3**	CMML 1	46, XY, del(20)(q11q13) [20]	nd	no	nd	no	**p.Arg166X**	no	nd
38	CMML 1	46, XY [20]	normal-like	no	nd	no	no	no	no
52	CMML 2	46, XX, inv(11)(p15q22) [20]	normal-like	no	nd	no	no	no	no
**90**	CMML 2	46, XX, t(1;3)(p36;q21) [20]	normal-like	no	nd	no	p. =	no	no
8	CMML 2	46, XY [20]	normal-like	no	nd	no	no	no	nd
25	CMML 1	46, XY [20]	normal-like	no	nd	no	**p.Leu56Ser**	no	nd
**63**	CMML 2	46, XY [20]	normal-like	no	nd	no	no	no	no
74	CMML 1	46, XY, del(20)(q11q13) [20]	20q11-q13 loss, 13q14 loss (RB1)	no	nd	no	no	no	no
87	CMML 1	46, XY [20]	normal-like	no	nd	no	**p.Arg320X**	no	no

34	**AT-CMML**	46, XX [20]	normal-like	no	nd	**p.Gln510His**	no	no	**USP16_E1-RUNX1_E7**
37	**AT-CMML**	46, XY, del(20)(q11q13) [13]/46, idem, -7, +mar [5]/46, idem, del(12)(p11-12p13) [5]	20q11-q13 loss, 7p loss, 7q11-q21 loss, 7q31-qter loss, 7q21 gain (CDK6)	no	nd	no	**p.Arg166X**	nd	No
80	**AT-CMML**	46, XY, del(9)(q21q34) [19]/46, XY [20]	9q21.11-q22.33 loss, 17q11 loss (NF1)	nd	**no**	no	no	no	No
88	**AT-CMML**	47, XY, +8 [11]/46, XY [9]	Tri 8, 21q21 losses (RUNX1)	no	nd	no	no	no	**USP16_E1-RUNX1_E5**
96	**AT-CMML**	46, XY, del(20)(q11q13), +mar [18]	20q11-q13 loss	no	nd	nd	no	no	no
106	**AT-CMML**	46, XY [20]	7 loss, 8q24 loss (MYC)	no	nd	nd	no	no	no

CMMLs are classified by a double line as in Additional file 1.

CMML: chronic myelomonocytic leukemia.

AT-CMML: acute transformation of CMML in acute myeloid leukemia (all M4 FAB type).

Samples 3 and 37 are from the same patient.

In bold: myeloproliferative CMML.

nd: not done.

E: exon.

Tri: trisomy.

### Mutations of RAS and RUNX1 genes

We analyzed the sequences of the three *RAS *genes. No mutation of *HRAS *was found. *NRAS *mutations were found in cases 12 and 78, and *KRAS *mutations in cases 79 and 89 (Table 1). One of these mutations affected codon 146 in coding exon 3, a rare type of *RAS *mutation that has been found in 4% colorectal cancers and two hematopoietic cell lines [13]. For patient 79 we determined that the mutation was present in a heterozygous state in the CD34-purified fraction of the BM cells, in the polynuclear neutrophils, monocytes and B lymphocytes but absent in the T cells (data not shown).

We examined the sequence of exons 3 and 13 of the *PTPN11 *gene. Mutations were found in three cases. No mutation was found in exon 7 of *RAF1*, which is a hotspot for mutations in Noonan syndrome (NS) [14,15]. *SOS1 *and *BRAF *were also sequenced in their most frequently mutated regions (exons 7–11 and kinase-encoding exons, respectively). One mutation was identified in *SOS1 *in a region involved in NS [16], none in *BRAF*. No mutation was found in *SPRED1 *[17].

The *NF1 *gene was analyzed for mutations in cases 79 and 80. A silent, so far unreported point mutation (c.2178G>C) was found in case 79 (Table 1). The deletion of an *RB1 *allele was confirmed by sequencing in case 74 and the remaining *RB1 *allele was normal. There was no JAK2 p.Val617Phe mutant in our panel of CMML cases.

**Figure 2:**
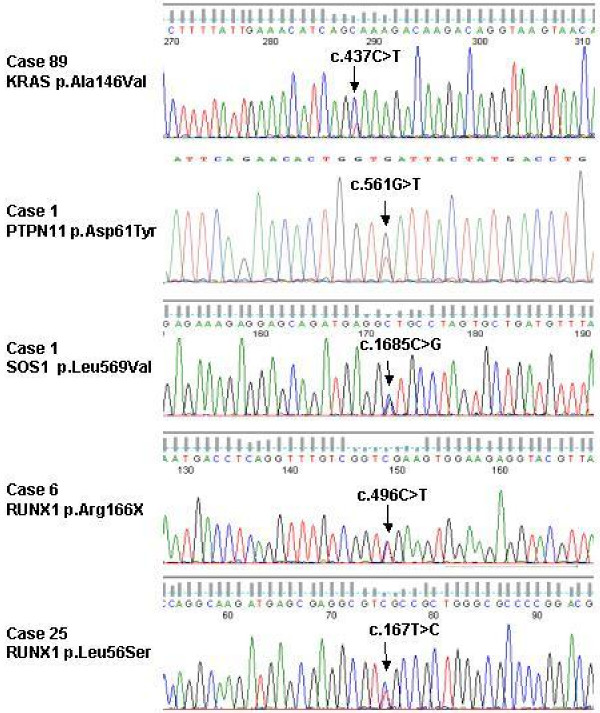
**Mutation of *RAS*, *PTPN11 *and *RUNX1 *genes in CMML**. Examples of mutations in candidate genes. From top to bottom, sequence of the mutated *KRAS, PTPN11, SOS1 *and *RUNX1 *alleles, demonstrating base change in the forward sequence at the position indicated by an arrow. The corresponding sequence is shown above. Primers and conditions used are described in additional file 2.

**Figure 3:**
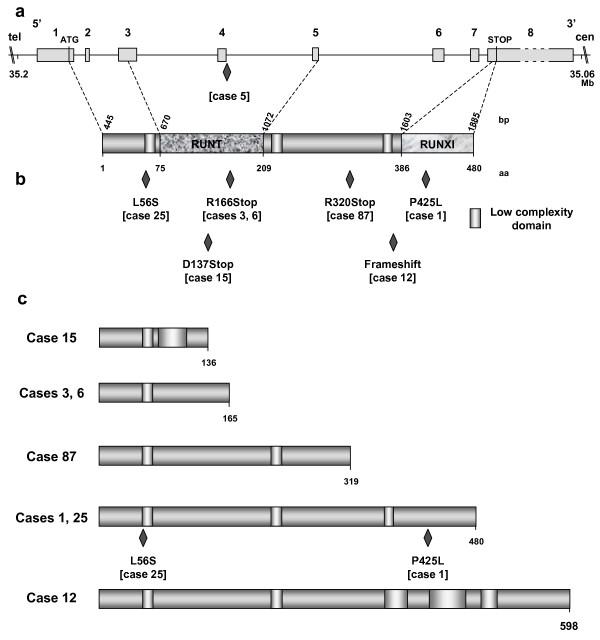
**Characterization of *RUNX1 *mutations in CMML patients**. A: Genomic organization of *RUNX1 *gene at 21q22.12 and RUNX1 protein. Functional (i.e. RUNT and RUNXI [for RUNX Inhibitor domain], as defined by PFAM accession numbers PF00853 and PF08504, respectively) and motifs of the RUNX1 protein were positioned according to the SMART program . Nucleotide (cDNA level) and deduced aminoacid sequences of the RUNX1 protein are positioned above and below the corresponding protein, respectively. The genomic *RUNX1 *sequence of CMML 5 exhibited a mutation in the consensus splicing sequence of intron 3. B: Mutations of *RUNX1*. All mutations but one introduced an aberrant stop codon (cases 3, 6, 12, 15 and 87). Two missense mutations (cases 1 and 25) were also observed. The mutations are located with respect to the modified aminoacid of the RUNX1 protein. C: Representation of putative mutated RUNX1 proteins. According to the SMART program, all putative modified proteins have lost their RUNT and RUNXI domains.

Mutations were found in the *RUNX1 *gene in 10 patients (30%). Mutation in case 90 is predicted to induce neither amino acid change nor splicing effect and thus was not considered as functionally deleterious. The nine other nucleotide variations would result in truncated or mutant proteins. *RUNX1 *mutations are described in Figures 2 and 3.

Finally, no mutation was found in the *STK11/LKB1 *and *SYK *kinase genes.

### A novel, cryptic rearrangement of RUNX1 following inv(21q)

**Figure 4:**
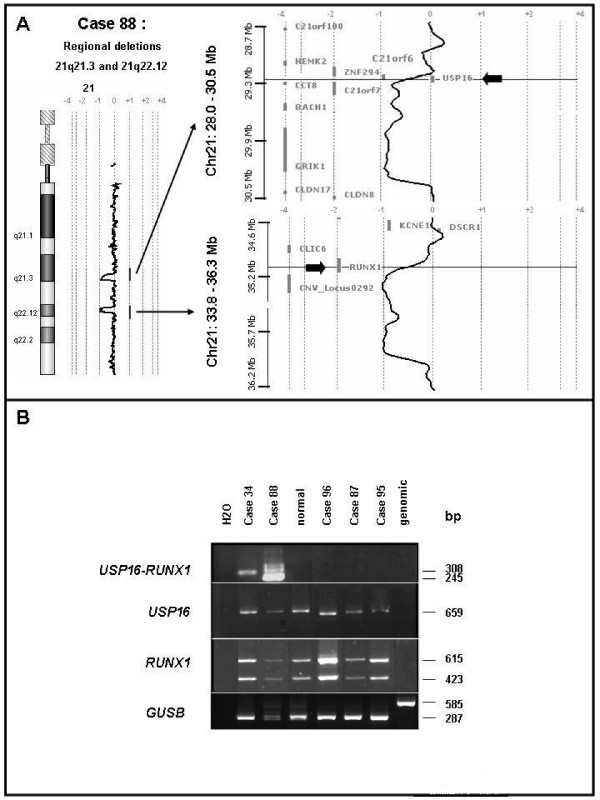
**Genomic rearrangements involve *USP16 *and *RUNX1 *genes in CMML patients**. A: CMML 88 aCGH profile of chromosome 21 shows regional deletions in 21q21.3 and 21q22.12. Arrows point to *USP16 *and *RUNX1 *genes targeted by transition profiles located in these respective regions. This suggests that potential gene breaks involve *USP16 *and *RUNX1*. B: PCR characterization of *USP16-RUNX1 *fusions in CMML. *USP16, RUNX1 *and *USP16-RUNX1 *transcripts were detected in the BM cells of the patients. The size of amplified products is shown on the right. The existence of alternatively spliced *RUNX1 *products could explain the various sizes observed for *USP16-RUNX1 *and *RUNX1 *transcripts. cDNA of normal lymphocytes were used as control. Primers specific for the human *GUSB *transcript were used for control of the RT-PCR quality control.

**Figure 5:**
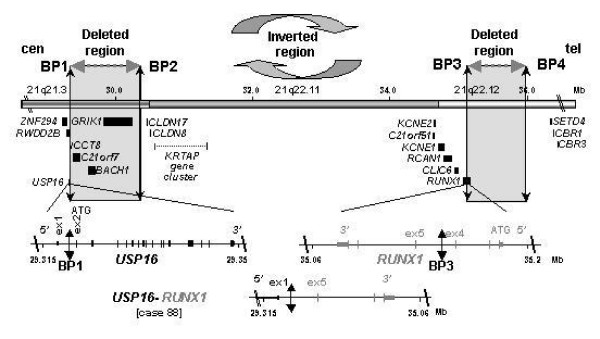
***USP16-RUNX1 *rearrangement in CMML 88**. Organization of chromosomal region 21q21.3-q22.12 with the location of the breakpoints (BP) and deleted regions from centromere (cen) to telomere (tel). Mb scale of the corresponding 21q21.3, 21q22.11 and 21q22.12 regions corresponds to cytogenetic bands. Only genes flanking affected regions are reported on the figure. Breakpoints BP1 and BP3 targeting *USP16 *and *RUNX1 are *associated with deletions defined by intervals [BP1-BP2] and [BP3-BP4]. The *USP16-RUNX1 *gene fusion is explained by the inversion of the central interval [BP2-BP3]. ATG codons are in exon 2 (ex 2) and exon 1 (ex 1) of *USP16 *and *RUNX1*, respectively.

The aCGH profile of case 88 showed two losses at 21q21.3 and q22.12 of about 1.04 Mb and 0.82 Mb, respectively (Figure 4A). They spanned the 3' part of *USP16*, including exons 2 to 19, *CCT8*, *BACH1 *and *GRIK1 *as well as the 5' part of *RUNX1 *(including exons 1 to 4), respectively. We hypothesized that such a peculiar pattern could be due to a cryptic inv(21)(q21q22) associated with a microdeletion at one of the breakpoints. Given the features and orientation of the various potentially-involved genes, we surmise that a fusion could involve *RUNX1 *and *USP16 *(encoding a de-ubiquitinating enzyme). This was confirmed by nested PCR amplification of reverse-transcribed RNA from the patient's BM cells, which detected a 245 bp-long *USP16-RUNX1 *transcript (Figure 4B). No reciprocal transcript was detected. Sequence analysis showed that the result of the inversion/fusion generated a chimeric *USP16-RUNX1 *transcript. The break/fusion was not present in the germline since we did not find the *USP16-RUNX1 *transcript in buccal smear cells of the patient. The *USP16-RUNX1 *gene fuses exon 1 of *USP16 *to exon 5 of *RUNX1 *thus not preserving the canonical ATG codons. The chimeric transcript exhibited several stop codons in its 5' part but the presence of multiple ATG codons through exons 5 to 7 of *RUNX1 *sequence could be used as new start codons and generate putative truncated RUNX1 proteins. A similar *USP16-RUNX1 *fusion (without microdeletion) was found in CMML 34 (Table 1). In the two cases, the *USP16-RUNX1 *fusion transcripts did not have an open reading frame using the canonical start codons of *USP16 *or *RUNX1 *(Figure 5). According to the SMART program , functional domains (i.e. RUNT and RUNXI [for RUNX Inhibitor domain], as defined by PFAM accession numbers PF00853 and PF08504, respectively) should disappear in such putative truncated RUNX1 proteins. RUNT and RUNXI domains are encoded mainly by exons 3 to 5 and exon 8, respectively (Figure 3). The partial conservation of *RUNX1 *transcript sequence (exons 5 to 8) and a new folding could explain conformational changes and the absence of RUNT and RUNXI domains.

In total, *RUNX1 *was altered by mutation (9) or break (2) in 11 patients (8 CMMLs and 3 AT-CMMLs) (Table 2).

**Table 2:** Summary of results.

	**All CMMLs ** **(N = 30)**	**All non AT-CMMLs ** **(N = 24)**	**MP-CMMLs ** **(N = 13)**	**MD-CMMLs ** **(N = 11)**	**AT-CMMLs ** **(N = 6)**	***RUNX1 *** **alteration**
**Normal-like**	17	16	6	10	1	7
**Trisomy 8**	3	2	2	0	1	3
**Del20q**	4	2	1	1	2	3
**RAS pathway mutation**	7	6	6	0	1	4
***RUNX1 *alteration**	11	8	5	3	3	11
***RUNX1 *mutation**	9	8	5	3	1	9
***USP16-RUNX1***	2	0	0	0	2	2

RAS pathway mutations include point mutations in KRAS, NRAS and PTPN11. *RUNX1 *alterations include *RUNX1 *mutations and breaks. Two cases (1 AT and 1 non-AT) are from the same patient.

Unsurprisingly, the 11q inversion in case 52 and the balanced t(1;3)(p36;q21) in case 90 escaped aCGH detection. The 11q inversion was probably a case of *NUP98-DDX10 *fusion [18] and the t(1;3) a case of *PRDM16/MEL1-RPN1 *fusion [19].

### Different alterations in MP- and MD-CMML

Excluding the 6 AT-CMMLs, *RAS *and *PTPN11 *mutations were found in 6 of the 13 MP-CMMLs (~46%) whereas no such mutation was found in the 11 MD-CMMLs (Table 2). In contrast, *RUNX1 *mutations occurred in both MP- (5 cases) or MD-CMML (3 cases).

## Discussion

We have established the first high resolution genome profiling of CMML and found a high frequency of *RAS *and *RUNX *gene alterations.

### CMML and the RAS pathway

In the majority of cases the aCGH profiles did not show any alteration. This suggests that rearrangements and copy number aberrations are not prominent in CMML and that aCGH is only in part suited for obtaining further insight into the pathogenesis of this disease. However, in a small proportion of the cases aCGH was informative, pointing to known tumor suppressor genes such as *NF1 *and *RB1*. However, neither gene was mutated in the remaining allele. Deletion of *NF1 *was particularly interesting since it led us to suspect an alteration of the RAS pathway and a similarity with juvenile myelomonocytic leukemia (JMML). JMML is a chronic myelomonocytic disease that occurs early in life, often on a genetic background of NS, and neurofibromatosis type 1. [20-22]. Half of NS are caused by germline mutations in the *PTPN11 *gene, which encodes a RAS pathway-regulating phosphatase. Germline mutations in *KRAS*, *SOS1, RAF1, BRAF *and other genes of the RAS pathway account for the other NS cases [14,15,20,23]. Syndromes caused by a hyperactivation of the RAS pathway also include Costello, cardio-facio-cutaneous (CFC), hereditary gingival fibromatosis and LEOPARD syndromes, and are collectively called neuro-cardio-facial-cutaneous (NCFC) syndromes [24].

We thus sequenced genes coding for proteins involved in the RAS signaling pathway and a *RAS *mutation was found in four cases (~14%), including a codon 146 mutation in *KRAS*. In a recent study of CMML, mutations of *KRAS *and *NRAS *genes were found in 9 patients out of 32 [25]; two of the six KRAS mutations were in codon 146. It is thus possible that mutation at this site is more frequent than expected, at least in hematopoietic diseases. Germline mutations of *KRAS *have been found in NCFC syndromes [24,26]. Germline *RAS *mutations induce precursor lesions and especially myeloproliferative disorders that resemble JMML and CMML [27-29]. In patient 79 the KRAS p.Gly12Ser substitution was present in the myeloid and B lymphoid lineages but not in T cells. We found mutations of *PTPN11 *exon 3 in 2 cases and exon 13 in one case (other exons were not screened). Somatic mutations of *PTPN11 *occur in approximately one-third of JMMLs but are less frequent in CMMLs [30,31]. Our combined results indicate that mutations in the RAS pathway occurred in at least one-fourth of CMML cases. No *HRAS *mutation was detected, in agreement with the absence of hematological manifestations in *HRAS*-linked Costello syndrome. CMML (at least MP-CMML) shares molecular features with JMML, i.e. similar non-specific alterations of chromosomes 7, 8 and 20, gene fusions [32] and alterations of genes suggesting activation of the RAS pathway. Inhibitors of the RAS pathway might be efficient in treating CMML [33,34].

### CMML and RUNX1 alterations

Two cases showed a break in *RUNX1 *due to an inversion of chromosomal region 21q21-22. *RUNX1 *encodes the DNA-binding, alpha subunit of the core binding factor (CBF) and is viewed as a tumor suppressor gene whose haplo-insufficiency or dominant-negative mutations play a role in leukemogenesis [35,36]. The gene is also frequently involved in translocations, with more than 15 different partners [37,38]. CBFB is the β subunit of the heterodimeric CBF factor. CBF regulates hematopoietic stem cell behavior and is essential for definitive hematopoiesis [39]. We show here that *RUNX1*, already known as a major translocation breakpoint, may even be more frequently altered than thought. Indeed, the 21q inversion is not detectable by karyotyping and, if not for the interstitial microdeletion, would not be detected by aCGH.

*RUNX1 *mutations were frequent in our series of CMML. In contrast to RAS pathway mutations, mutations of *RUNX1 *have been reported unfrequently in CMML and JMML, perhaps due to the experimental approach [40]. Overall, we found alterations of *RUNX1 *in roughly half of the non-acutely transformed cases. They resulted in various truncated or aberrant proteins that could act as dominant-negative isoforms or result in haplo-insufficiency.

Case 12 shows a deletion of *CALN1*, encoding calneuron 1, a calmodulin-like protein. Calmodulin regulates calcineurin, which is recruited by RUNX1 to regulate granulocyte-macrophage colony-stimulating factor [41]. Finally, CDK6, whose gene is amplified in case 3, inhibits RUNX1 activity [42]. Noticeably, amplification of *CDK6 *has been recently described in lymphoma [43]. Thus, alteration of RUNX1 function may occur frequently and by different mechanisms in CMML.

### Other alterations

Patient 52 had been treated for breast cancer and the CMML may be due to a therapy-related pericentric inversion of chromosome 11 with *NUP98-DDX10 *fusion. CMML 90 may be due to a fusion between *PDRM16 *(*EVI1*-like) and *RPN1*. Such fusion is found in MDS and AML-M4 [44].

Not surprisingly, CMML shares molecular features with MDS and AML, especially therapy-related diseases, including loss or partial deletions of chromosome 7, rearrangement of the *RUNX1 *gene, mutations of *RAS *and *PTPN11 *[45].

### Cooperative and exclusive alterations

RAS pathway mutations and *RUNX1 *alterations were not mutually exclusive. *RAS *mutations, *PTPN11 *mutations and *NF1 *deletion were mutually exclusive. However, in case 1, mutations of *PTPN11 *and *SOS1 *were found. The two mutations could synergize but the *SOS1 *mutation has never been reported and its functional relevance remains unknown.

### Myeloproliferative vs myelodysplastic CMML

Six RAS pathway alterations were found in 13 MP-CMMLs but none in 11 MD-CMMLs (p = 0.016, Fisher exact test). An even higher proportion of MP-CMMLs may be due to mutation in the RAS pathway because other cases could be due to mutations occurring elsewhere in these genes or in other genes of the RAS pathway. This suggests that MP- and MD-CMMLs could develop along two different oncogenic pathways, specific of two distinct diseases. This hypothesis reinforces our previous observation on CMML gene expression [8]. However, in a recent study *RAS *mutations were distributed independently of the white blood cell count [25].

*RB1 *deletion, *RUNX1 *mutation and inv(11)(p15q22) were the only identified alterations in our series of MD-CMML. These alterations are neither specific of CMML nor of MD-CMML (p = non significant) since we found several (5/13) alterations of *RUNX1 *in MP-CMMLs. Thus, the molecular biology of MD-CMML remains unclear. Yet, we now know that MD-CMML shares *RUNX1 *alteration with other diseases. It is tempting to speculate that *RUNX1 *alterations are responsible for the dysplasia whereas RAS pathway mutations are responsible for the myeloproliferation. In terms of treatment, CMMLs with a RAS pathway mutation may benefit from drugs able to target the RAS/RAF/MAPK pathway [33,34] (e.g. sorafenib), while all CMMLs may benefit from therapy restoring RUNX function.

## Conclusion

We have identified two important features of the molecular biology of CMML: i) – RAS pathway mutations are involved, at least, in roughly half of MP-CMML; ii) – *RUNX1 *alterations are frequent in CMML; they may result from mutations or chromosome rearrangements. Importantly, RAS and RUNX1 alterations are not exclusive, showing that, already, two oncogenic hits may coexist at this chronic stage.

## Competing interests

The authors declare that they have no competing interests.

## Authors' contributions

VGB, CA, DS, NV and MJM selected the cases and provided and reviewed the clinical and biological data. VT, NA, VR, SP, CH, MBA and SO generated the sequencing data. JA, VT and MC generated the aCGH data. JA discovered and studied the USP16-RUNX1 fusion. VGB, MJM, DB and MC supervised the study and wrote the manuscript. All authors approved the study and the manuscript.

## Pre-publication history

The pre-publication history for this paper can be accessed here:



## Supplementary Material

Additional File 1 **Clinical and hematological features of the studied CMMLs.** This table provides details on disease and patients. CMMLs are classified by a double line according to antecedents. Samples 3 and 37 are from the same patient. CMML: chronic myelomonocytic leukemia. In bold: myeloproliferative CMML and corresponding WBC. AT-CMML(AML-M4): acute transformation of CMML in acute myeloid leukemia of M4 FAB type. MDS: myelodysplasic syndromes. RARS: refractory anemia with ring sideroblasts. EPO: erythropoïetin. WBC: white blood cell count. TA: therapeutic abstention. DGP: dysgranulopoiesis, DMK: dysmegakaryocytopoiesis, DEP: dyserythropoiesis. 2, 95, 75: hematological malignancies were concomitant of the diagnosis of CMML. 8*: thrombopenia with a diagnosis of MDS (not done in our Institute). Apparition of anemia 2 weeks before the sampling and then treated with EPO. Variable monocytosis around 1.2 G/L but unlikely below 1 G/L at the time of sampling. 25*: persistant and stable monocytosis since June 2006 (1.2 G/L) but unlikely below 1 G/L at the time of sampling. 63*: mild anemia and thrombopenia with a diagnosis of MDS (not done in our Institute) and then under surveillance. 74*: treated for AML (not in our Institute) in 1992 (chemotherapy) and relapse (AML-M5) in our Institute in 2005 (chemotherapy). The cytological aspect of the bone marrow in January 2007 was CMML type 2. 34*: because of the important bone marrow dysplasia and the imprtant cellularity the diagnosis of a acute phase of CMML was made. 80*: antecedent of monocytosis non explored. 88*: thrombopenia since 2005 and diagnosis of CMML (2006) made out of our Institute. 106*: stable monocytosis since February 2000. One sister with breast cancer. One brother with head and neck cancer and her mother with colon cancer (deceased). Click here for file

Additional File 2 **Conditions of DNA sequencing used in this study for various genes.** the table provides information (primers, PCR conditions) on sequence experiments conducted in this study. Click here for file

Additional File 3 **Primers used for PCR detection of *RUNX1 *alteration.** The table provides information of primers used to detect USP16-RUNX1 fusion. Click here for file
