# Supplementary material for: Genome profiling of chronic myelomonocytic leukemia: frequent alterations of RAS and RUNX1 genes
Source: BMC Cancer. 2008 Oct 16;8:299. doi: 10.1186/1471-2407-8-299 (PMC2588460; doi:10.1186/1471-2407-8-299)
Supplement: Additional File 2 — Conditions of DNA sequencing used in this study for various genes. the table provides information (primers, PCR conditions) on sequence experiments conducted in this study. [file 1471-2407-8-299-S2.pdf]

Supplementary Table 2 : Conditions of DNA sequencing used in this study for various genes.

Amplicon	Sequence oligonucleotide: Forward 5'-3'	Sequence oligonucleotide: Reverse 5'-3'	Annealing	Size PCR product (bp)	PCR conditions	Sequencing oligonucleotide
BRAF exon 11	TCCCTCTCAGGCATAAGGTAA	CGAACAGTGAATATTTCCTTTGAT	60℃	313	Hot Star Taq Qiagen	F + R
BRAF exon 15	TCATAATGCTTGCTCTGATAGGA	GGCCAAAAATTTAATCAGTGGA	60℃	224	Hot Star Taq Qiagen	F + R
JAK2 exon 12	CTCCTCTTTGGAGCAATTCA	TATCGCAACTCCCAAGTTCTC	55℃	496	Hot Star Taq Qiagen	internal oligo TGAATA GTCCTACAGTGTTTTCAGTTT
RAF1 exon 7	GGTTGTACAGGTAGAGTTTGCCC	TCCTTGATCAGATTTGAAACCC	54℃	363	Hot Star Taq Qiagen	F + R
HRAS exon 1	GGAGACCCTGTAGGAGGACC	GAGGAAGCAGGAGACAGGG	60℃	282	Hot Star Taq Qiagen	R + F
HRAS exon 2	ATGAGGGGCATGAGAGGTACCAG	CATCCAGGACATGCGCAGAGA	60℃	377	Hot Star Taq Qiagen	F + R
HRAS exon 3	TCTCTGCGCATGTCCTGGAT	TAGCCCCACTAAGACTCAGAACCA	60℃	619	Hot Star Taq Qiagen	F + R
HRAS exon 4	AAGGCTTGATCCCACAGCA	ACCTCCATGTCCTGAGCTTGT	60℃	340	Hot Star Taq Qiagen	F + R
KRAS exon 1	GACTGAATATAAACTTGTGG	CTCTATTGTTGGATCATAT	45℃	109	Ampli Taq Gold Applied Biosystem	F + R
KRAS exon 2	GCCATTTGTCCGTCATCTTTGGAG	TGCATGGCATTAGCAAAGACTCA	55℃	415	Hot Star Taq Qiagen + MgCl2	F + R
KRAS exon 3	AGGAAGGAAAATTTGGTGTAGTGGA	AGAAGCAATGCCCTCTCAAGAGAC	54℃	452	Hot Star Taq Qiagen	F + R
KRAS exon 4a	CTTGCACATGGCTTTCCCAGTA	AGTGGTTGCCACCTTGTTACCT	54℃	272	Hot Star Taq Qiagen	F + R
KRAS exon 4b	CCTGTACACATGAAGCCATCGT	CTAACAGTCTGCATGGAGCAGGAA	54℃	299	Hot Star Taq Qiagen	F + R
NRAS exon1	TGTAGATGTGGCTCGCCAAT	TGATCCGACAAGTGAGAGAC	55℃	228	Hot Star Taq Qiagen	F + R
NRAS exon 2	GGCAATAGCATTGCATTCCC	TCCCTAGTGTGGTAACCTCA	55℃	344	Hot Star Taq Qiagen	F + R
RUNX1 exon 1	GCTGAAACAGTGACCTGTCTTGG	AGCTGCCATTTCATTACAGGCAAAGC	58℃	219	Hot Star Taq Qiagen	F + R
RUNX1 exon 2	CTCCAGTGCTAAAAAGTGTAAG	CATATACACATCTATGAAGGTGTGTAC	58℃	212	Ampli Taq Gold Applied Biosystem	internal oligo GTCGGGGAAAGTTAATT
RUNX1 exon 3	CGGCTTGTTGTGATGCGTATCC	ACCACCCTCTCCGGGCCAGTA	58℃	304	Ampli Taq Gold Applied Biosystem + Q solution (Qiagen)	F + R
RUNX1 exon 4	CTGCATTTGTCCTTTGACTGGTG	GTGGGTTTGTTGCCATGAAACGTG	58℃	239	Hot Star Taq Qiagen	F + R
RUNX1 exon 5	CAGGCCACCAACCTCATTCTGTT	GCCCCAAGTGGATGCACTTAC	58℃	173	Hot Star Taq Qiagen	F + R
RUNX1 exon 6	CAATACTTTTTCTGATCTCTTCCCT	TGGGCTCCATCTGGTACTTACCC	58℃	286	Ampli Taq Gold Applied Biosystem	R + internal oligo GCAGAAACTAGATGATCAGAC
RUNX1 exon 7	CCCACCCCACTTTACATATAATTGACC	CCCAGCTCAGCTGCAAAGAATGTG	58℃	246	Hot Star Taq Qiagen	F + R
RUNX1 exon 8a	CTCACTTCCGCTCCGTTCTCT	GGCCGAGGCGCCGTAGTACAGG	58℃	301	Ampli Taq Gold Applied Biosystem + Q solution (Qiagen)	F + R
RUNX1 exon 8b	CCAGCTCGCCCTCCTACCA	TTGTCGCGAACAGGAGGCCC	63℃	329	Ampli Taq Gold Applied Biosystem + Q solution (Qiagen)	F + R
SOS1 exons 7 and 8	ATTGTGCTCGCATAGTCGTG	AAATTCACACTTGAATATGTTACAAA	60℃	426	Hot Star Taq Qiagen	F (ex7) + R (ex8)
SOS1 exon 9	TGCTCCCCATTTCTTTTCAG	CTTGAGGAGGGAACTGGGAT	60℃	500	Hot Star Taq Qiagen	F + R
SOS exon 10	CATGAGCTCTAGGTTTTCTGTCA	GCCAAGTGACCTCATTTTCTC	60℃	950	Hot Star Taq Qiagen	F + internal oligo GA TGACACCAATGAATACAAGCA
SOS1 exon 11	GCAGTGCATTACCAAGTCCA	TTGTTCACTGACAAGTTCCATTTT	60℃	591	Hot Star Taq Qiagen	F + R
SPRED1 exon2	GGTACCGTTCTGGGTGAGG	AAGTTTCGGATGGGTCTGG	59℃	269	Hot Star Taq Qiagen	F + R
SPRED1 exon3	AAACACCTTAGTCACCACATGTTA	TGCCTTTAACACAGAAACAGC	55℃	431	Hot Star Taq Qiagen	F + R
SPRED1 exon4	AGCGTTGTATCACCTCAGTTTG	TGAGGTTTCAAAGCCTGGTC	55℃	398	Hot Star Taq Qiagen	F + R
SPRED1 exon5	TTAATTGCCAGGCAGTCCAG	ATGAGGGATGCTCAACCTGT	55℃	340	Hot Star Taq Qiagen	F + R
SPRED1 exon6	CATTTGAGTTTTGGGAATTGCT	GGGCAGATGGTTTTAACGTC	55℃	398	Hot Star Taq Qiagen	F + R
SPRED1 exon7	GGGGGAAATGATTCTATTTTTATTC	CAGCGTTTAAGCACATAGGC	55℃	396	Hot Star Taq Qiagen	F + R
SPRED1 exon8-5'	TGCTTTCCTCATAGTCCACCA	GGAGCATCCTGACATTTTCC	55℃	552	Hot Star Taq Qiagen	F + R
SPRED1 exon8-3'	AGACGCAGCCTTCCTCATTA	AGTTAGGCATGGCGTGAAAC	55℃	569	Hot Star Taq Qiagen	F + R
STK11 exon 1	ACAATCGTTTCTGTTGGAAG	CCAGCTCAGGGTGTTAAGAG	55℃	579	Hot Star Taq Qiagen	F + R
STK11 exon 2	CACTGTGAACTCACAGCTTCTC	AGCCATTGCCACAATGGCTGACTTC	55℃	390	Hot Star Taq Qiagen	F + R
STK11 exon 3	CCTGAGCTGTGTGTCCTTAGCG	ATATCAGGACAAGCAGTGTG	55℃	212	Hot Star Taq Qiagen	F + R
STK11 exons 4 and 5	GTGTGGCTCCCTGCTGGACCTAGCCTTTC	TACATTTCCATAAAGTAAGCACCCCCTA	55℃	667	Hot Star Taq Qiagen	F + R
STK11 exon 6	CGTCAACCACCTTGACTGACCACG	CCTGCTGGGGTCTCGGGGCCAATAG	55℃	289	Hot Star Taq Qiagen	F + R
STK11 exon 7	TTAGGAGCGTCCAGGTATCACCC	ACCCCCACCACGCCCTGCTCTAGCG	55℃	271	Hot Star Taq Qiagen	F + R
STK11 exon 8	GAGGACATGGCTGAGCTTCTGTGGT	AAGCTGTCCTTGTTGCAGACA	55℃	443	Hot Star Taq Qiagen	F + R
STK11 exon 9	CAGCATTTCAGGCTGGATACACCT	ACGGTCACCATGACTGACTA	55℃	465	Hot Star Taq Qiagen	F + R
