# Supplementary material for: Genome profiling of chronic myelomonocytic leukemia: frequent alterations of RAS and RUNX1 genes
Source: BMC Cancer. 2008 Oct 16;8:299. doi: 10.1186/1471-2407-8-299 (PMC2588460; doi:10.1186/1471-2407-8-299)
Supplement: Additional File 3 — Primers used for PCR detection of RUNX1 alteration. The table provides information of primers used to detect USP16-RUNX1 fusion. [file 1471-2407-8-299-S3.doc]

# Additional file 3: Primers used for PCR detection of *RUNX1* alteration

| **Amplified**  **products** | **PCR**  **type** | **Primers combinations** | |
| --- | --- | --- | --- |
| RUNX1 wt | nested | *RUNX1*_591F  5’- CAGGCAAGATGAGCGAGGCG -3’ | *RUNX1*_1498R  5’- GCCTGGATAGTGCATGCGGG-3’ |
|  |  | *RUNX1*_738F  5’- TCTGCTCCGTGCTGCCTACG -3’ | *RUNX*1_1352R  5’- AAATGGGCGTTGCTGGGTGC -3’ |
| ***USP16 wt*** |  | *USP16*_54F  5’- TCTGGCTTCGACTCCGTCGC-3’ | *USP16*_712R  5’- TGGCTTTGGAGTTGTAATGCTGGC-3’ |
| ***GUSB*** |  | *GUSB*_1111F  5’-CCACGGTGTCAACAAGCATGAGG-3’ | *GUSB*_1398R  5’-GGGTGGTTCTTGTCCCTACGC-3’ |
| **USP16-RUNX1** | nested | *USP16*_54F  5’- TCTGGCTTCGACTCCGTCGC-3’ | *RUNX1*_1498R  5’- GCCTGGATAGTGCATGCGGG-3’ |
|  |  | *USP16*_89F  5’-AGGAGGAAGACGGAGCTGGC-3’ | *RUNX1*_1422R  5’-TATGGGCTCTGTCGCCGTGG-3’ |
| RUNX1- USP16 | Hemi nested | *RUNX1*_591F  5’- CAGGCAAGATGAGCGAGGCG -3’ | *USP16*_712R  5’- TGGCTTTGGAGTTGTAATGCTGGC-3’ |
|  |  | *RUNX1*_738F  5’- TCTGCTCCGTGCTGCCTACG -3’ | *USP16*_712R  5’- TGGCTTTGGAGTTGTAATGCTGGC-3’ |
